# Missed opportunities of HIV pre-exposure prophylaxis in France: a retrospective analysis in the French DAT’AIDS cohort

**DOI:** 10.1186/s12879-019-3915-5

**Published:** 2019-03-25

**Authors:** C. Lions, O. Cabras, L. Cotte, T. Huleux, A. Gagneux-Brugnon, A. Makinson, A. Cabié, B. Bonnet, C. Duvivier, L. Hocqueloux, E. Cua, A. Cheret, L. Hustache-Mathieu, V. Obry-Roguet, C. Jacomet, I. Poizot-Martin, the DAT’AIDS STUDY GROUP, C. Drobacheff-Thiébaut, A. Foltzer, K. Bouiller, L. Hustache-Mathieu, C. Chirouze, Q. Lepiller, F. Bozon, O. Babre, P. Muret, H. Laurichesse, O. Lesens, M. Vidal, N. Mrozek, C. Aumeran, O. Baud, V. Corbin, P. Letertre, S. Casanova, J. Prouteau, C. Jacomet, B. Hoen, I. Lamaury, I. Fabre, E. Curlier, R. Ouissa, K. Schepers, C. Herrmann-Storck, N. Dournon, D. Merrien, P. Perré, T. Guimard, O. Bollangier, S. Leautez, M. Morrier, F. Ader, F. Biron, A. Boibieux, L. Cotte, T. Ferry, P. Miailhes, T. Perpoint, S. Roux, S. Degroodt, C. Brochier, F. Valour, C. Chidiac, C. Dhiver, M. Saadia Mokhtari, A. Ménard, H. Tissot Dupont, C. Toméi, L. Meddeb, A. Y. Belkhir, I. Ravaux, S. Brégigeon, O. Zaegel-Faucher, V. Obry-Roguet, H. Laroche, M. Orticoni, M. J. Soavi, P. Geneau de Lamarlière, E. Ressiot, M. J. Ducassou, I. Jaquet, S. Galie, A. Galinier, P. Martinet, M. Landon, A. S. Ritleng, A. Ivanova, C. Debreux, C. Lions, I. Poizot-Martin, S. Abel, L. Cuzin, Nguyen S. Pierre-François, J. Pasquier, M. Pircher, K. Rome, B. Rozé, A. Cabié, N. Atoui, V. Le Moing, A. Makinson, N. Meftah, C. Merle de Boever, B. Montes, A. Montoya Ferrer, J. Reynes, M. André, L. Boyer, M. P. Bouillon, M. Delestan, T. May, C. Allavena, C. Bernaud, E. Billaud, C. Biron, B. Bonnet, S. Bouchez, D. Boutoille, C. Brunet-Cartier, C. Deschanvres, N. Hall, T. Jovelin, P. Morineau, V. Reliquet, H. Hue, S. Sécher, M. Cavellec, A. Soria, V. Ferré, E. André-Garnier, A. Rodallec, M. Lefebvre, O. Grossi, O. Aubry, F. Raffi, P. Pugliese, S. Breaud, C. Ceppi, J. Courjon, E. Cua, J. Cottalorda, P. Dellamonica, E. Demonchy, A. De Monte, J. Durant, C. Etienne, S. Ferrando, J. G. Fuzibet, R. Garraffo, A. Joulie, K. Risso, V. Mondain, A. Naqvi, N. Oran, I. Perbost, S. Pillet, B. Prouvost-Keller, C. Pradier, S. Wehrlen-Pugliese, V. Rio, E. Rosenthal, S. Sausse, G. Zouzou, L. Hocqueloux, T. Prazuck, C. Gubavu, A. Sève, M. Niang, C. Boulard, A. Cheret, C. Goujard, Y. Quertainmont, E. Teicher, N. Lerolle, D. Vittecoq, O. Deradji, F. Fourreau, C. Pallier, A. Barrail-Tran, R. Landman, V. Joly, C. Rioux, S. Lariven, A. Gervais, F. X. Lescure, S. Matheron, F. Louni, C. Godard, Z. Julia, M. Chansombat, D. Rahli, C. Mackoumbou-Nkouka, C. Charpentier, D. Descamps, G. Peytavin, Y. Yazdanpanah, P. H. Consigny, G. Cessot, P. Bossi, J. Goesch, J. Gilquin, G. Benabdelmoumen, F. Lanternier, C. Charlier, K. Amazzough, B. Henry, B. Pilmis, C. Rouzaud, M. Morgand, F. Touam, C. Louisin, C. Duvivier, O. Lortholary, R. Guery, F. Danion, J. Lourenco, P. Parize, N. Etienne, M. Launay, C. Rouzioux, V. Avettand Fenoel, M. A. Valantin, F. Caby, R. Tubiana, R. Agher, S. Seang, L. Schneider, R. PaLich, C. Blanc, C. Katlama, J. L. Berger, Y. N’Guyen, D. Lambert, D. Lebrun, I. Kmiec, M. Hentzien, V. Brodard, F. Bani-Sadr, E. Botelho-Nevers, A. Gagneux-Brunon, A. Frésard, F. Lucht, P. Fischer, M. Partisani, C. Cheneau, M. Priester, M. L. Batard, C. Bernard-Henry, E. de Mautort, D. Rey, M. Alvarez, N. Biezunski, A. Debard, C. Delpierre, P. Lansalot, L. Lelièvre, G. Martin-Blondel, M. Piffaut, L. Porte, K. Saune, P. Delobel, F. Ajana, I. Alcaraz, V. Baclet, A. Boucher, P. Choisy, T. Huleux, B. Lafon-Desmurs, H. Melliez, A. Meybeck, A. Pasquet, M. Pradier, O. Robineau, N. Viget, M. Valette

**Affiliations:** 1 APHM Hôpital Sainte-Marguerite, Service d’Immuno-hématologie clinique, Aix-Marseille University, Marseille, France; 2 Service des maladies infectieuses et tropicales, CHU Bichat, Paris, France; 3 Service des maladies infectieuses et tropicales, Hospices Civils de Lyon, Lyon, France; 4 Service Universitaire des maladies infectieuses et du voyageur, CH Tourcoing, Tourcoing, France; 5 Service d’Infectiologie, CHU Sainte-Etienne, Groupe Immunité des Muqueuses et Agents Pathogènes, Institut Presage, Université de Lyon, Lyon, France; 6 Infectious and Tropical Diseases Department, University Hospital Montpellier, Montpellier, France; 7 UMI 233/INSERMU1175, IRD, University Montpellier, Montpellier, France; 8 Service des maladies infectieuses et tropicales, CHU de Martinique, INSERM CIC 1425 and Université des Antilles EA 4537, La Martinique, France; 9 Maladies Infectieuses et tropicales, CHU HOTEL DIEU, Nantes, France; 10 APHP-Hôpital Necker-Enfants Malades, Service de Maladies Infectieuses et Tropicales, Centre d’infectiologie Necker-Pasteur, F-75015 Paris, France; 11 Institut Pasteur, Centre Médical de l’Institut Pasteur, Centre d’infectiologie Necker-Pasteur, F-75015 Paris, France; 12 Université Paris Descartes, Sorbonne Paris Cité, Equipe d’Accueil EA 7327, F-75015 Paris, France; 13 IHU Imagine, F-75015 Paris, France; 14 Service des maladies infectieuses et tropicales, CHR d’Orléans –La Source, Orléans, France; 15 Service des maladies infectieuses et tropicales, CHU de Nice, Nice, France; 16 Service de Médecine Interne, CHU Kremlin Bicêtre, AP-HP, Kremlin Bicêtre, France; 17 Service des maladies infectieuses et tropicales, CHU Besançon, Besançon, France; 18 Service des maladies infectieuses et tropicales, CHU Clermont Ferrand, Clermont Ferrand, France; 19 Aix-Marseille Univ, INSERM, IRD, SESSTIM, APHM Sainte-Marguerite, Clinical Immuno-Hematological Unit Marseille, Aix Marseille Univ, Marseille, France; 20 Immuno hematological Unit/ service d’Immuno- hématologie Clinique, Centre d’Informations et de Soins de l’Immunodéficience Humaine et des Hépatites virales, 270 boulevard de Sainte Marguerite, 13009 Marseille, France

**Keywords:** Pre-exposure prophylaxis, PrEP, HIV, Missed opportunities, HIV testing

## Abstract

**Background:**

HIV pre-exposure prophylaxis (PrEP) was implemented in France in November 2015 based on individual-level risk factors for HIV infection. We evaluated the proportion of missed opportunities for PrEP among newly HIV-diagnosed people entering the Dat’AIDS cohort in 2016.

**Methods:**

Multicenter retrospective analysis in 15 French HIV clinical centers of patients with a new diagnosis of HIV infection. Among them we differentiated patients according to the estimated date of infection: those occurring in the PrEP area (a previous negative HIV test in the last 12 months or those with an incomplete HIV-1 western blot (WB) with no HIV-1 anti-Pol-antibody at time of HIV diagnosis) and those in the pre-PrEP area (older infections). Epidemiological, biological and clinical data at HIV diagnosis were collected. Clinicians retrospectively identified potential eligibility for PrEP based on individual-level risk factors for HIV infection among those infected in the PrEP area.

**Results:**

Among 966 patients with a new HIV diagnosis, 225 (23.3%) were infected in the PrEP area and 121 (53.8%) had complete data allowing evaluation of PrEP eligibility. Among them, 110 (91%) would have been eligible for PrEP, median age 31 years, with 68 (75.6%) born in France and 10 (11.1%) in Central/West Africa, with more than one previous STI in 19 (15.7%). The main eligibility criteria for PrEP were being a man who had sex with men or transgender 91 (82.7%) with at least one of the following criteria: unprotected anal sex with ≥2 partners in the last 6 months: 67 (60.9%); bacterial sexually transmitted infection in the last 12 months: 33 (30%); Use of psychoactive substances in a sexual context (chemsex): 16 (14.5%). PrEP was indicated for other HIV risk factors in 25 (22.7%).

**Conclusion:**

With 91% (110/121) of patients infected in the PrEP area eligible for PrEP, this study highlights the high potential of PrEP in avoiding new infection in France but also shows a persistent delay in HIV testing. Thus, an important limit on PrEP implementation in France could be insufficient screening and care access.

## Background

The HIV epidemic remains active in France, as evidenced by approximately 6000 new HIV infectionsThe development of a concentrated diagnosed yearly since 2011 [1, 2] despite a favorable cascade of care [3]. The dynamic of the epidemic is mainly driven by men who have sex with men (MSM) (44% of new HIV diagnosis) but also by the migrant women’s population (23%), mainly from sub-Saharan African countries [1]. Several actions have been taken to curb the dynamics of the epidemic across the territory, of whom pre-exposure prophylaxis treatment (PrEP) with 300 mg daily tenofovir (TDF) co-formulated with 200 mg emtricitabine (FTC). HIV pre-exposure prophylaxis is an effective tool in preventing HIV infection among high-risk men who have sex with men. Indeed, a PrEP uptake at 25% of a high-risk population of MSM, without any additional preventative strategies could prevent 30.7% of infections [4]. PrEP was first evaluated in France with the ANRS Ipergay trial who was initiated in July 2014 among 6 clinical centers in France. Then, PrEP was implemented since November 25, 2015, first as part of a Temporary Recommendation for Use (TRU) [5] based on individual-level risk factors for HIV infection, followed by a marketing authorization extension without advance fees for the patient in February 2017 [6].

French guidelines assist clinicians in the evaluation of patients who are seeking PrEP, in commencing and monitoring patients on PrEP including PrEP dosing schedules, management of side-effects and toxicity, use of PrEP in pregnancy and in chronic hepatitis B infection and how to cease PrEP. Daily PrEP can be used continuously or for shorter periods of time, or on demand [7]. According to French guidelines, two negative HIV tests performed 4 weeks apart without HIV risk behavior during these 4 weeks are required [8] in order to avoid PrEP initiation during the HIV seroconversion period.

However, since the introduction of this preventive tool in France, new HIV infections are still occurring [9]. It is still too early at that time to quantify the impact of PrEP on the number of new infections, and to date, no data are available on the proportion of newly HIV-infected people who would have been potential candidates for PrEP in France. We report here the results of a retrospective study evaluating the proportion of missed opportunities for PrEP among newly HIV-infected people during the year 2016.

## Methods

This multicenter retrospective analysis was performed in the French Dat’AIDS Cohort (NCT 02898987 ClinicalTrials.gov). This cohort represents a collaboration between 21 major French HIV clinical centers using a common electronic medical record (NADIS®) [10] for the follow-up of HIV infected adults coinfected or not with hepatitis B virus (HBV) and hepatitis C virus (HCV), among which data from 59,829 patients were collected during the year 2016. For this retrospective study, 15 centers accepted to participate. The data collection has been approved by the French National Commission on Informatics and Liberty (CNIL 2001/762876; MR 003: 2044467 v.0), and all patients signed an informed consent before being included in this database. Patient-related data are recorded during medical encounters in a structured database, allowing for clinical, epidemiological or therapeutic studies. Data quality is ensured by automated checks during data capture, regular controls, annual assessments, and ad hoc processes before any scientific analysis is performed.

### Data collection

Socio-demographic characteristics (age, sex, birth area, HIV transmission route), lifetime history of other sexually transmitted infections (STI) (e.g., syphilis, gonorrhea, chlamydia) and biological data (HIV viral load, CDC stage, CD4 T cell count, hepatitis C, hepatitis B, hepatitis A and syphilis serology) were collected at the time of HIV diagnosis.

We also collected past HIV test and western blot (WB) at diagnosis. We differentiated patient according to estimate date of infection: before availability of PrEP or after. Then, among diagnosis occurring in 2016, we considered patients infected in the PrEP area, those with a previous negative HIV test realized in the last 12 months or those with an incomplete HIV-1 WB with no HIV-1 anti-Pol-antibody at time of HIV diagnosis (primary infection). Patients who did not presented these characteristics (older or indeterminate date of infection) were considered as infected in the pre-PrEP area.

For patients infected in the PrEP area, at each center, the referring clinician of each patient retrospectively identified eligibility for PrEP according to the individual-level risk factors for HIV infection as defined by TRU criteria (see Table 3 for TRU criteria).

### Statistics

We firstly compared patient’s characteristics according to the estimated date of infection (PrEP area versus pre-PrEP area). Secondly, we compared patient’s characteristics according to PrEP eligibility. Continuous and categorical variables were summarized as the median and interquartile range [IQR] or frequency and percentage, respectively. For these comparisons we used the non-parametric Mann-Whitney test for continuous variables and Chi^2^ or Fisher’s exact test for categorical data. All statistical analyses were performed using SAS software version 9.3 (SAS Institute Inc., Cary, North Carolina).

## Results

In 2016, 966 patients were newly diagnosed as HIV seropositive in the 15 HIV clinical centers. Most of them were male (*N* = 709; 73.4%), HIV transmission route was MSM for 46.7% (*N* = 449) and heterosexual intercourse for 42.7% (*N* = 410) (Table 1). This last category concerned mostly persons from sub-Saharan Africa (*N* = 177; 43.0%) and especially women (*N* = 217; 53.0%) (data not shown). Among the 966 patients, 225 patients fulfilled the criteria of infection occurring in the PrEP area, of whom 108 were in primary infection.

**Table 1:** Patients’ characteristics at the time of HIV diagnosis according to the estimated date of infection (pre-PrEP area versus PrEP area)

Description *N* (%) or Median [IQR]	Pre-PrEP area infections *N* = 741	PrEP area infections *N* = 225	Total *N* = 966	*p*
Socio-demographic characteristics				
Age (years)	37 [29–47]	33 [26–44]	36 [29–46]	0.001
Sex				
Male	507 (68.42)	202 (89.78)	709 (73.40)	*<.0001*
Female	234 (31.58)	23 (10.22)	257 (26.60)	
Birth area				
West or central Africa	213 (34.98)	20 (11.30)	233 (29.64)	*<.0001*
France	238 (39.08)	128 (72.32)	366 (46.56)	
Other	158 (25.94)	29 (13.37)	187 (23.79)	
HIV transmission route				
MSM	281 (37.97)	168 (76.02)	449 (46.72)	*<.0001*
Heterosexual	365 (49.32)	45 (20.36)	410 (42.66)	
Other	94 (12.70)	8 (3.62)	102 (10.61)	
CDC Stage				
A	490 (78.03)	194 (96.04)	684 (82.41)	*<.0001*
B	37 (5.89)	7 (3.47)	44 (5.30)	
C	101 (16.08)	1 (0.50)	102 (12.29)	
Biological data at HIV-diagnosis				
HIV viral load (log cp/ml)	4.92 [4.05–5.60]	5.50 [4.45–6.40]	5.20 [4.30–6.00]	<.0001
CD4 (cell count/mm^3^)	347 [155–520]	480 [334–635]	378 [202–555]	*<.0001*
CD4 (cell count /mm^3^)				
≤200	205 (30.73)	14 (6.60)	219 (24.91)	*<.0001*
200–350	135 (20.24)	46 (21.70)	181 (20.59)	
350–500	145 (21.74)	59 (27.83)	204 (23.21)	
>500	182 (27.29)	93 (43.87)	275 (31.29)	
HCV antibodies				
Negative	565 (96.42)	189 (97.93)	754 (96.79)	*0.30*
Positive	21 (3.58)	4 (2.07)	25 (3.21)	
HBV AgHbs				
Negative	570 (95.80)	195 (98.98)	765 (96.59)	*0.03*
Positive	25 (4.20)	2 (1.02)	27 (3.41)	
HVA antibodies				
Negative	179 (37.45)	105 (63.25)	284 (44.10)	*<.0001*
Positive	299 (62.55)	61 (36.75)	360 (55.90)	
Syphilis serology				
Negative	340 (79.63)	98 (65.33)	438 (75.91)	0.0004
Positive	87 (20.37)	52 (34.67)	139 (24.09)	
History of other sexuality transmitted disease (STI): *N* = 348				
Syphilis				
No	177 (91.24)	110 (71.43)	287 (82.47)	<.0001
Yes	17 (8.76)	44 (28.57)	61 (17.53)	
Gonorrhea				
No	187 (96.39)	134 (87.01)	321 (92.24)	0.001
Yes	7 (3.61)	20 (12.99)	27 (7.76)	
Chlamydia				
No	191 (98.45)	141 (91.56)	332 (95.40)	0.002
Yes	3 (1.55)	13 (8.44)	16 (4.60)	
Number of STIs				
0	160 (82.47)	92 (59.74)	252 (72.41)	*<.0001*
1	29 (14.95)	40 (25.97)	69 (19.83)	
≥2	5 (2.58)	22 (14.29)	27 (7.76)	

### PrEP area vs pre-PrEP area infected patients

Compared to the patients infected in the PrEP area, those infected in the pre-PrEP area were significantly older and were more often women from west or central Africa and infected by heterosexual intercourse. The two groups also differed significantly according to CDC stage C with a higher percentage found in the pre-PrEP area group (16.1% vs. 0.50%, *p* < 0.0001), and positive syphilis serology at the time of HIV diagnosis, less often found in the pre-PrEP area group (20.4% vs. 34.7%, *p* = 0.0004). Median CD4 cell count/mm^3^ (347 [155–520] vs. 480 [334–635], p < 0.0001) and HIV viral load in log/ml (4.92 [4.05–5.60] vs. 5.50 [4.45–6.40], *p* < .0001) were significantly lower in the pre-PrEP area group. History of STI was available for 348 patients, and the percentage of those with a previous STI (chlamydia and the number of previous STIs) was significantly lower in those infected in the pre-PrEP area (Table 1).

### Eligible for PrEP vs non-eligible among patients infected in the PrEP area

Among the 225 patients infected in the PrEP area, individual-level risk factors for HIV infection were available for 121 patients (53.7%), among which 110 (90.9%) patients would have been eligible for PrEP. The patients’ characteristics are reported in Table 2. Patients with available data of individual-level risk factors for HIV infection were not different from those without these data (*n* = 104; data not shown) except for age, with patients with available data being significantly younger than the others (31 [25–41] vs 36 [29–45] years; *p* = 0.02).

**Table 2:** Characteristics of patients infected during the PrEP area according to eligibility for PrEP

N (%) or Median [IQR]	Not eligible for PrEP (*n* = 11)	Eligible for PrEP. (*n* = 110)	PrEP area infection (*n* = 121)	*p*
Socio-demographic characteristics				
Age (years)	31 [29; 38]	31 [25; 44]	31 [25; 41]	0.74
<25 years	1 (9.09)	25 (22.73)	26 (21.49)	0.61
[25–35]	6 (54.55)	41 (37.27)	47 (38.84)	
[35–45[	3 (27.27)	18 (16.36)	21 (17.36)	
[45–55[	1 (9.09)	18 (16.36)	19 (15.70)	
≥55 years	0 (00)	8 (7.27)	8 (6.61)	
Sex				
Male	6 (54.55)	104 (94.55)	110 (90.91)	0.001^a^
Female	5 (45.45)	6 (5.45)	11 (9.09)	
Birth area				
West or central Africa	1 (11.11)	9 (11.11)	10 (11.11)	0.85
France	6 (66.67)	62 (76.54)	68 (75.56)	
Other	2 (22.22)	10 (12.35)	12 (13.33)	
HIV transmission route				
MSM	4 (36.36)	91 (85.85)	95 (81.20)	0.001^a^
Heterosexual	6 (54.55)	13 (12.26)	19 (16.24)	
Other	1 (9.09)	2 (1.89)	3 (2.56)	
Primary HIV infection				
No	6 (54.55)	53 (48.18)	59 (48.76)	*0.76*
Yes	5 (45.45)	57 (51.82)	62 (51.24)	
Biological data at HIV diagnosis				
HIV viral load (log/ml)	5.6 [4.7; 5.9]	5.4 [4.4; 6.4]	5.4 [4.3; 6.3]	0.64
CD4 (cell count /mm3)	470 [340; 650]	478 [328; 635]	480 [340; 641]	0.46
CD4 (%)	19 [14–25]	26 [20–34]	25 [19–33]	0.05
HCV antibodies				
Negative	9 (100.00)	93 (97.89)	102 (98.08)	*1* ^a^
Positive	0 (0.00)	2 (2.11)	2 (1.92)	
HBV AgHbs				
Negative	9 (100.00)	93 (97.89)	102 (98.08	*1* ^a^
Positive	0 (0.00)	2 (2.11)	2 (1.92)	
HAV antibodies				
Negative	6 (100.00)	51 (66.23)	57 (68.67)	*0.17* ^a^
Positive	0 (0.00)	26 (33.77)	26 (31.33)	
Syphilis serology				
Negative	7 (100.00)	40 (60.61)	47 (64.38)	*0.05*
Positive	0 (0.00)	26 (39.39)	26 (35.62)	
History of other sexuality transmitted diseases (STI)				
Syphilis				
No	10 (90.91)	77 (70.00)	87 (71.90)	*0.18*
Yes	1 (9.09)	33 (30.00)	34 (28.10)	
Gonorrhea				
No	10 (90.91)	96 (87.27)	106 (87.60)	*1* ^a^
Yes	1 (9.09)	14 (12.73)	15 (12.40)	
Chlamydia				
No	11 (100.00)	99 (90.00)	110 (90.91)	*0.60* ^a^
Yes	0 (0.00)	11 (10.00)	11 (9.09)	
Number of STIs				
0	10 (90.91)	62 (56.36)	72 (59.50)	0.06^a^
1	0 (0.00)	30 (27.27)	30 (24.79)	
≥2	1 (9.09)	18 (16.36)	19 (15.70)	

^a^Fisher’s exact test

Among the 121 patients, the median [IQR] age was 31 years [25–44], without a significant difference according to PrEP eligibility (Table 2). The age distribution was also similar: 21.5% of patients were under 25 years old, 38.8% between 25 and 35, 17.4% between 35 and 45, 15.7% between 45 and 55 and 6.6% over 55 years. The birth area was France in 75% of cases and Central/West Africa in 11%, without a significant difference between the two groups. A history of STI was reported in 15.7% and was also similar between the groups. However, in the group of patients eligible for PrEP, there were significantly more MSM and more patients with positive syphilis serology at the time of HIV diagnosis.

### Distribution of PrEP eligibility criteria among patients eligible for PrEP

The distribution of PrEP eligibility criteria are presented in Table 3. The most common situation was MSM who had condomless anal intercourse with at least two different men or transgender partners in the last 6 months. Among MSM, use of psychoactive drugs during sexual intercourse was reported in 14.5%. In 23% of cases, PrEP was indicated for other substantial risk factors for HIV (prostitution with unprotected sexual intercourse, vulnerability, physical factors increasing HIV risk transmission).

**Table 3:** Distribution of PrEP eligibility criteria as defined by Temporary Recommendation for Use (TRU) in France in 2016 [5] among the 110 patients eligible for PrEP*

Men who have sex with men or transgender and at least one of these criteria	91 (82.7)
- Unprotected anal intercourse with at least two different partners in the last 6 months	67 (60.9)
- Incident of sexually transmitted infection (STI) in the last 12 months	33 (30.0)
- Several uses of Post-Exposure Prophylaxis in the last 12 months	1 (0.9)
- Use of psychoactive drugs during sexual intercourse	16 (14.5)
Other persons who are at substantial risk of HIV acquisition among whom PrEP can be considered case by case	25 (22.7)
- Person in situation of prostitution submitted to non-protected sexual intercourse	4 (3.6)
- Person in a situation of vulnerability exposed to non-protected sexual intercourse with a person belonging to a group with a high HIV prevalence: ✓ native of a high HIV prevalence area ✓ who has multiple sexual partners ✓ who injects drug	13 (11.8) 8 (7.3) 4 (3.6) 1 (0.9)
- Person who has non-protected sexual intercourse with persons with physical factors increasing HIV risk transmission to the exposed person (anal or genital ulceration, associated STI, bleeding)	3 (2.7)
- Other situations judged at high risk of HIV acquisition by sexual way: ✓ Migrant in precarious situation, probably exposed to non-consensual sex ✓ HIV seropositive partner not previously detected ✓ HIV seropositive regular partner (treated with no HIV viral load data) ✓ Untreated HIV seropositive partner ✓ MSM reports with excessive alcohol use during non-protected sexual intercourse	5 (4.5) 1 (0.9) 1 (0.9) 1 (0.9) 1 (0.9) 1 (0.9)

*several indications can be found for one patient

## Discussion

With 225 infections occurring in the PrEP area and 741 in the pre-PrEP-area reported in 15 French major HIV clinical centers during the year 2016, this retrospective study highlights the high number of infections that could have been avoided with PrEP, estimated to be 91% among our population. Moreover, this study confirms the ongoing HIV epidemic in France that is driven mainly by recent infections among MSM. These results also stress the need to reinforce HIV screening strategies in order to decrease the number of missed opportunities and delay for HIV testing in all populations at risk.

To our knowledge, only one previous study looked at missed opportunities for PrEP among new HIV diagnoses in South Carolina [11]. They found that African American/Black, women and younger people were more likely to have had health care visits prior to their HIV diagnosis, which was used as a proxy for a missed opportunity. However, they did not study missed opportunities with regard to risky practices and recommendations, and new diagnoses were approximated by CD4 count at diagnosis ≥500 cells/ mm^3^. Therefore, our study is the first that evaluated missed opportunities of PrEP in a large cohort of newly HIV infected patients in France. However, to date, it is unknown how effective PrEP is on the dynamic of epidemic in the context of other implemented HIV prevention strategies, including condom use, seroadaptative practice, and treatment as prevention (TasP) [4]. Recently, Brown et al. reported a decrease by 32% in new HIV diagnoses among MSM at selected London sexual health clinics between October 2015 through September 2016. Although this decrease could be attributed to higher testing volumes and rapid initiation of treatment after diagnosis (TasP), these preliminary data suggest that a combination of PrEP and TasP could make elimination of HIV transmission achievable [12].

The development of a concentrated HIV epidemic among MSM has been observed in several countries on different continents, including Asia, Africa and South America [13–15]. In our study, among the 225 infections occurring in the PrEP area, 76% were MSM. If considering only the 121 patients with an infection occurring in the PrEP area and available information concerning PrEP eligibility, the estimated proportion of patients eligible for PrEP at the time of HIV infection was 91% and concerned mainly MSM.

Of note, use of psychoactive drugs during sexual intercourse was reported by 14.5% of the patients eligible for PrEP. A significant increase of psychoactive substances use in a sexual context (chemsex), and some measures of HIV-related behaviors (condomless sex with two or more partners in the past 3 months, self-reported bacterial STI diagnosis in the past year, Post-Exposure Prophylaxis use in the past year and HIV testing in the past 6 months) among HIV-negative MSM has been reported in the last few years [16]. Chemsex disclosure in sexual health settings was reported to be associated with a high level of HIV infection risk [17] and higher rates of STI diagnoses, including hepatitis C [18, 19], which concerned 2 patients in our study. Of note, for those patients who are going to initiate antiretroviral therapy, they should be advised of the high risk of potential drug-drug interactions between some recreational drugs and some antiretrovirals agents, with the major risk concerning ritonavir-boosting or cobicistat-boosting agents and some nonnucleoside reverse transcriptase inhibitors [20, 21]. Clinicians should evaluate the risk of such interactions before initiating HIV treatment and consider antiretroviral regimens with a lower risk of drug interactions [22].

Among eligible patients for PrEP, syphilis serology was positive at the time of HIV diagnosis in 39.4%, and a history of syphilis, gonorrhea, and chlamydia was present in 30.0, 12.7 and 10.0% of patients, respectively. Moreover, a history of two or more STIs affected 16.4% of these patients. High rates of STI have been reported among PrEP users, as well as high rates of condomless sex and increasing rates of STI over time [23, 24]. Recently, an increase in the rate of STI in PrEP users was reported in a study conducted in Montreal, Canada [25]. These data raised the discussion of how PrEP may impact STI control efforts. However, since we found more STIs in PrEP area HIV-infected patients, our data suggest that STIs should also be considered as a driver of HIV transmission risk among MSM. Thus, as suggested by Scott et al., expanded PrEP implementation among high-risk MSM could promote better control of STIs through the systematic screening recommended before PrEP initiation and during the follow-up of PrEP users [26].

Before taking PrEP, users should also be tested for hepatitis B, to prevent reactivation or reinfection in case of PrEP interruption, even if this phenomenon seems to not be very common [27, 28]. Furthermore, for patients with negative HBV serology, HBV vaccination should be administered [7, 29]. In our study, chronic hepatitis B was diagnosed in two patients.

With 21% of patients under 25 years old and 7% over 55 diagnosed in the PrEp area, and with 22.7% of our patients presenting with high-risk situations for HIV infection other than MSM intercourse, our data highlight the need for diverse information campaign targets in order to optimize access to PrEP for different population groups. Indeed, PrEP may impact the HIV epidemic, but only if it reaches the at-risk population [30].

By July 2017, 5352 patients had initiated a tenofovir-emtricitabine treatment for PrEP in France [31]. Among them, they were mostly men, with a median age of 37 years, 9.2% were between 16 and 25 years, and 5% were over 55 years. The gap between availability and usage of PrEP by the population of concern is challenging and asks about obstacles of generalized use and ways for improving the spread of this preventative tool. To date, studies of factors influencing attitudes and behaviors towards PrEP have been established among high-risk populations but not among newly infected persons.

Factors associated with missed opportunities for PrEP can be of individual, social or structural kinds [32]. Major factors highlighted in the literature are mostly on the individual level and concern the intention to take PrEP but not use of PrEP. Moreover, the roles played by physicians, networks, communities and policies are also paramount for enhanced PrEP usage. The design of our study did not allow us to evaluate these factors, and further studies on the obstacles and facilitators of PrEP use are needed.

The proportion of MSM and west or central African women populations among those with new diagnoses observed in our study (40 and 15%, respectively) was consistent with those previously reported in France in 2015: 43% were MSM and 23% migrant women [1]. Among them, Pre-PrEP area infection that is a proxy of delay in HIV testing concerned 77% of our population, of whom 35% were born in west or central Africa, 31% with CD4 cell count less than 200/mm^3^ and 20% between 200 and 350/mm^3^. The delay in HIV diagnosis remains a persistent problem in France as in other countries [33, 34] leading to negative clinical, economic and public health implications. Late presentation is associated with increased patient morbidity and mortality and limits the effectiveness of all subsequent steps in the cascade of HIV care [35, 36]. We did not collect the conditions of HIV testing in our study, so we could not determine if the late diagnoses were due to low risk perception and/or lack of awareness about HIV. Whatever the reason, our results stress the need to reinforce HIV screening strategies in our country, taking into account specific populations more hidden and less engaged in concerned networks.

### Limits

Our study had several limits. First, the study was retrospective and the selection criteria involved under-estimation of infection occurring during the PrEP area among new HIV diagnoses. Indeed, among the 741 patients considered infected during the pre-PrEP area, 198 patients did not have a serology in the last 12 months nor an incomplete WB so we could not exclude that these were infected in the PrEP area. However, in our study, the estimated proportion of infections occurring in the last 12 months was 23%, similar to the one-third of recent infections (in the 6 months) found in 2015 in France [1]. Second, we can also not exclude information biases because some physicians may have more often prospectively responded to the patient’s eligibility for PrEP in cases of PrEP eligibility. Third, no behavioral data, like sexual practices, were collected and we cannot determinate if some specific behavior are linked to the PrEP eligibility nor some specific practices.

## Conclusion

This study highlights the high potential of PrEP in avoiding new infection in France, as 91% of patients infected in the PrEp area were eligible for. However, our results highlight a persistent delay in HIV testing in France as observed in other countries. PrEP could markedly decrease HIV infection if combined with a high diagnosis rate and viral suppression [37]. Thus, an important limit of PrEP implementation could be insufficient screening and care access. A prospective study on newly infected persons could allow to disentangle real obstacles of PrEP use in France and to optimize PrEP criteria.

## Abbreviations

HBV: Hepatitis B virus
HCV: Hepatitis C virus
IQR: Interquartile range
MSM: Men who have sex with men (MSM
PrEP: Pre-exposure prophylaxis (PrEP)
STI: Sexually transmitted infections
TasP: Treatment as prevention
TRU: Temporary Recommendation for Use (TRU
WB: Western blot
